# Multiple Spontaneous Intracranial-Extracranial Arterial Dissections in a Patient with Osteogenesis Imperfecta

**DOI:** 10.1155/2017/8520961

**Published:** 2017-07-02

**Authors:** Mehmet Kolukısa, Elif Gökçal, Azize Esra Gürsoy, Çiğdem Deniz, Ayşe Aralaşmak, Talip Asil

**Affiliations:** ^1^Neurology Department, Bezmialem Vakıf University, İstanbul, Turkey; ^2^Radiology Department, Bezmialem Vakıf University, İstanbul, Turkey

## Abstract

A 40-year-old male with osteogenesis imperfecta (OI) was admitted to the hospital with an acute right monoparesis. Diffusion-weighted MRI showed infarction in the territory of the left anterior cerebral artery (ACA) and in the left posterior cerebral artery (PCA). In his vascular imaging, occlusion of the left vertebral artery (VA) starting from V2 segment was consistent with dissection and pseudoaneurysm in the right ACA. We presented this case because of the presence of spontaneous and simultaneous occurrence of both intracranial and extracranial arterial dissections in OI.

## 1. Introduction

Dissection of the cranial and cervical arteries is a leading cause of ischemic stroke accounting for 20% of the strokes occurring in patients younger than 45 years of age [1]. Despite the occurrence of arterial dissection following minor or major trauma, dissections may also occur spontaneously [2]. Collagen vascular disease may play an important role in the pathogenesis of spontaneous arterial dissection, particularly when multiple vessels are involved [3–5]. Osteogenesis imperfecta (OI) is one of the connective tissue disorders associated with arterial dissections. However, there is no report presenting spontaneous arterial dissections in both intra- and extracranial arteries in OI.

Herein, we present a patient with osteogenesis imperfecta (OI) who presented with intra- and extracranial arterial dissections occurring simultaneously.

## 2. Case Presentation

**Figure 1:**
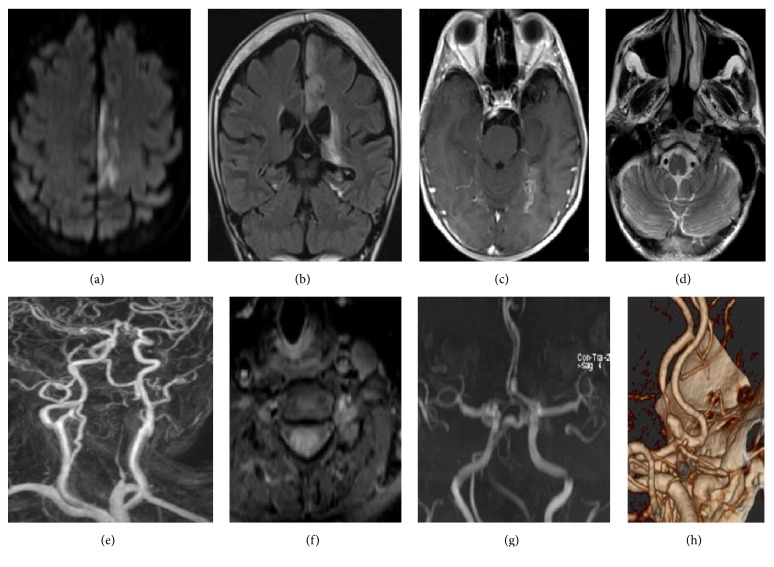
DWI showing (a) left acute anterior cerebral artery infarction and subacute posterior cerebral artery infarction in coronal FLAIR and (b) in postcontrast axial T1A (c); V4 segments of the vertebral arteries with a symmetrical appearance (d). Luminal narrowing along the left V2 in contrast-enhanced cervical MRA (e); hyperintensity in left V2 suggestive of intraluminal or intramural thrombus in fat-suppressed T1A (f); dissection presenting with narrowing and dilatation at the proximal part of the left A2 at 3D-TOF MRA (g) and volume rendering CTA (h). CTA was considered to be suggestive of dissected left V2, and the hyperintensity in T1A images was explained on the basis of intramural thrombus formation.

A 40-year-old male patient was admitted to the emergency department with a complaint of sudden onset weakness in his right leg starting after taking a bath. There was no antecedent trauma or recent infection. He had a diagnosis of OI clinically with the presence of recurrent fractures, short stature, blue sclera, and dental problems. He had no cerebrovascular risk factor and he took no medication. On admission, his blood pressure was 130/70 mmHg and heart rate was 71 bpm (regular), and body temperature was 36.8°C. There was no abnormality in his electrocardiography. The neck was supple and Kernig's sign was absent. Neurologically, he was alert and oriented with normal speech. Moderate motor monoparesis of his right leg with an extensor plantar response has been found. There was no cranial nerve dysfunction, sensory disturbances, or cerebellar dysfunction. Other neurological findings were normal, as were his complete blood count, liver and kidney function tests, blood glucose, electrolytes and thyroid function tests, and vasculitis panel. Cranial computed tomography scanning disclosed no area of abnormal density. His brain magnetic resonance imaging (MRI) revealed acute cerebral ischemia in the territory of the left anterior cerebral artery (ACA) and subacute cerebral ischemia in the territory of the posterior cerebral artery (PCA) (Figure 1). On MR angiography, the left vertebral artery (VA) was occluded at V2 segment. A fat-suppressed cervical MRI showed crescent-shaped high-intensity signals in VA, consistent with the existence of intramural thrombus. Also, there was a pseudoaneurysm in the right ACA.

Based on these clinical and radiological characteristics, a diagnosis of artery-to-artery embolism caused by left VA dissection was suspected, and he was immediately started on a regimen of anticoagulant treatment along with hospitalization.

## 3. Discussion

Heritable disorders of connective tissue affecting the arterial wall are recognized in a minority of arterial diseases of the central nervous system, such as arterial dissections. In a previous study, 5% of the patients with spontaneous cervical arterial dissection have been found to have hereditary connective tissue disorders [6] while in another study involving 65 patients with dissections, only 3 had signs of connective tissue disorders, although the skin biopsy revealed connective tissue abnormalities in 55% of patients but no connective tissue abnormality has been reported in 10 healthy controls [7].

OI is a hereditary condition seen in 1/100,000 of the general population [8]. Type 1 represents the most common form occurring in 50% of the cases. The principal defect in type 1 OI is the deficiency or aberrant production of type 1 collagen, which consists of two alpha 1 and one alpha 2 polypeptide chains coded by COL1A1 and COL1A2 genes. Unsurprisingly, clinical manifestations are consistent with the distribution of type 1 collagen, which is the most abundant collagen in the human body. The main clinical manifestation is excessive bone fragility, blue sclerae, and hearing loss [9]. It may also be associated with alterations of vascular connective tissue resulting in abnormalities and malformations of vascular structures [1, 2]. A variety of forms of vascular involvement such as the dissection, cerebral aneurysms, carotid-cavernous fistulas, and moyamoya-like disease have been reported in patients with OI [8, 10–13].

The underlying pathogenesis responsible for spontaneous dissection is unknown. Recently, impaired vasomotion among patients with multiple spontaneous extracranial arterial dissections has been reported as the predisposing factor to dissection [14]. Collagen vascular disorders may play a role in the pathogenesis of spontaneous arterial dissection by a similar mechanism.

In a recent study, 22% of patients with spontaneous extra- and/or intracranial arterial dissections had multivessel arterial dissections [15]. In this study, patients with multiple dissections were younger and had spontaneous dissections, and the presence of pseudoaneurysm was higher.

In the literature, spontaneous bilateral dissection of internal carotid arteries has been reported in a patient with a mild phenotypic variant of OI having a point mutation in the cxl(1) chain of type I collagen [12]. And, recently, progressive bilateral vertebral artery dissection in a patient with OI has been reported [16].

To the best of our knowledge, there is no report presenting multiple and spontaneous occurrence of both intracranial and extracranial arterial dissections in OI. Our case has been presented due to the presence of arterial dissections in different arteries supplying different vascular regions. It is important to visualize both intra- and extracranial arteries for simultaneous dissections in stroke patients with OI.
